# Tick species diversity, seasonality and feeding behavior in small wild mammals in the northern foothills of the Dabie Mountains, China

**DOI:** 10.1016/j.ijppaw.2025.101171

**Published:** 2025-11-26

**Authors:** Yin Fu, Pitambar Dhakal, Zi Yan, Mengyao Yang, Chaofeng Ma, Yayun Wu, Jiahui Wang, Qinglin Wang, Longxian Zhang

**Affiliations:** a College of Veterinary Medicine, Henan Agricultural University, Zhengzhou, Henan, China; b International Joint Research Laboratory for Zoonotic Diseases of Henan, Zhengzhou, China; c Key Laboratory of Quality and Safety Control of Poultry Products, Ministry of Agriculture and Rural Affairs, Zhengzhou, Henan, China; d Technical Service Center for Animal husbandry and Veterinary Medicine of Xinyang, Henan, China

**Keywords:** Small wild mammals, Ticks, Seasonal activity, Species diversity, Host selectivity

## Abstract

Small wild mammals are major carriers of ticks in the field, yet their tick-carrying status in China remains inadequately studied. To access tick infestations on small mammals, we collected 1908 ticks from 267 rodents, 27 hedgehogs and 4 hog badgers in the northern foot of Dabie Mountain, China. We identified five tick species including: *Haemaphysalis hystricis*, *H. flava*, *H*. *longicornis*, *Ixodes granulatus*, and *Amblyomma testudinarium*. Notably, this represents the first recorded occurrence of *H. hystricis*, *I. granulatus*, and *A. testudinarium* in the study region, expanding their known geographical distribution, which can serve as evidence of its expansion towards the north China. The tick species exhibited distinct seasonal activity patterns: Adult *H*. *hystricis* demonstrated activity from June to August (July peak), the larval and nymphal were present from March through December, peaking in October and September separately. For *H. flava*, adults were found from April to September (August peak), while nymphs were recorded in May, July, and September (September peak), and Larvae were detected exclusively in July. Adult *H. longicornis* peaked in July (May–September). Adult *I. granulatus* were present from June to October (June peak), and nymphal appeared in March, October, and November (November peak), with larval restricted to March and November. The nymphal of *A. testudinarium* were collected during June and August (August peak). Ticks showed clear host preferences: The larvae and nymphs of *H. hystricis* exclusively infested rodents (particularly *Niviventer* and *Rattus*), while adults primarily parasitized hog badgers. Both *H. longicornis* and *H. flava* infested mainly burdens on hedgehogs. Moreover, the *I. granulatus*, and *A. testudinarium* only in rodents and hedgehogs separately. The study reminds us that ticks may be more widely distributed in Chinese wildlife, therefore, more attention needs to be paid to ticks on wild animals in the future.

## Highlights

• Ticks on wild small mammals show seasonal variation.
• Ticks have feeding preference to different kinds of small wild mammals.
• First reported *H. hystricis*, *I. granulatus*, and *A. testudinarium* in the study area.
• The evidence of *I. granulatus*, and *A. testudinarium* northward spread in China.

## Graphical abstract

**Figure:**
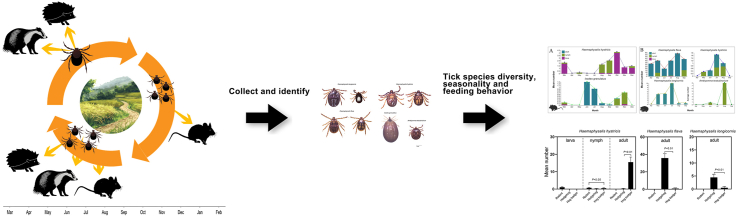

## 1 Introduction

Ticks, as obligate hematophagous ectoparasites, depend entirely on host blood for survival and reproduction. Their feeding behavior facilitates both mechanical and biological transmission of diverse tick-borne pathogens (TBP), including spirochetes, arboviruses, and apicomplexan protozoans (Zhong et al., 2024). The species composition and infestation intensity of ticks on animal hosts are governed by a complex interaction of ecological factors. Specifically, species with greater body surface area and wider activity ranges exhibited significantly elevated tick burdens, a consequence of both prolonged environmental exposure and increased host-vector encounter rates. Host specificity further influences infestation profiles. For instance, a previous study in Africa revealed that camel body surfaces hosted a significantly greater diversity of tick species compared to co-grazing livestock (goats, sheep, and cattle) (Makwatta et al., 2024). Notably, multi-host ticks exhibit stage-dependent host-switching strategies; larvae and nymphs of Ixodidae primarily parasitize small mammals, whereas adults preferentially infest large animals, including livestock and humans (Beldomenico et al., 2003; Zhang et al., 2019). Host phenological rhythms further modulate infestation dynamics, as evidenced by eastern rock sengis (*Elephantulus myurus*), which exhibit threefold higher larval loads during non-breeding seasons—a pattern linked to reduced grooming efficiency (Lutermann et al., 2012).

Wild animals are the primary hosts of ticks and play a central role in tick life cycles and transmission. The continued geographic expansion of tick species (Sonenshine, 2018) has garnered global concern, as migratory birds and other mobile hosts facilitate long-distance dispersal of both ticks and associated pathogens. These introduced populations may subsequently establish enzootic transmission cycles, often supported by local wild small mammal communities (Zhang et al., 2022a, 2023). China harbors remarkable tick biodiversity, with more than 120 documented species exhibiting distinct regional distributions shaped primarily by habitat characteristics (Zhang et al., 2019; Zhao et al., 2021). However, emerging evidence suggests that contemporary climate change and anthropogenic landscape modifications may significantly alter these distribution patterns, potentially enabling range expansions for several species in the near future (Yang et al., 2021). China's mammalian fauna exhibits a pronounced ecological imbalance, with large wild mammals occurring at exceptionally low densities while small mammals dominate wildlife communities (Zhao et al., 2022). This faunal composition significantly influences tick ecology, as small mammal-associated tick communities demonstrate marked regional heterogeneity in species diversity. Notably, northwestern China hosts the most diverse tick assemblages on small mammals, with Xinjiang and Tibet representing secondary hotspots of tick species richness (Hu et al., 2023; Li et al., 2024; Zhang et al., 2022b). While Central and Southern China also harbor diverse tick communities (Zhao et al., 2021), these regions remain understudied compared to other areas (Du et al., 2018).

Wild animals play a crucial role as hosts for field ticks and as natural reservoirs for tick-borne pathogens. Along the Mediterranean coast, 35 tick species and 17 wild animal hosts have been identified carrying up to 85 types of tick-borne pathogens, including bacteria, viruses, and parasites. The majority of these pathogens are traced back to game mammals like red deer and wild boar. Small wild mammals also carry various tick-borne pathogens, posing a significant transmission risk as they share habitats with humans and domestic animals. Hedgehogs have been found to harbor the Severe Fever with Thrombocytopenia Syndrome Virus (STFSV) throughout winter, with the *H. longicornis* tick on their bodies serving as a key vector for STFSV (Zhao et al., 2022). Additionally, hedgehogs and their ectoparasitic ticks can transmit several other pathogens, some of which are zoonotic, including Tick-borne encephalitis virus (TBEV), *Crimean-Congo hemorrhagic fever orthonairovirus* (CCHFV), *Bhanja bandavirus*, *Yersinia pestis*, *Rickettsia* spp., *Bartonella* spp., *Babesia* spp., *Candidatus* Neoehrlichia mikurensis, and *Leishmania* spp. (Benkacimi et al., 2023). Rodents, as significant vectors, not only impact public health security but also serve as reservoir hosts for tick-borne pathogens such as *Borreliella*, *Rickettsia*, and *Anaplasma* (Martello et al., 2019; Rymaszewska and Piotrowski, 2024; Xue et al., 2024). Comprehensive investigations of tick species composition and infestation patterns on small wild mammals are critical for several reasons: they enable accurate monitoring of tick distribution dynamics, inform predictive models of range expansions, and provide the ecological foundation for developing effective tick-borne disease (TBD) control strategies.

The northern foothills of the Dabie Mountains in central China have a mild temperate semi-humid monsoon climate, with notable seasonal fluctuations and annual precipitation over 1000 mm, which is primarily concentrated in the summer and autumn. The terrain is predominantly mountainous and hilly, with extensive forest coverage that sustains a rich diversity of wildlife. This location has been recognized as a hotspot for intermittent tick-borne incidents affecting both humans and livestock. Despite its epidemiological significance, the tick species parasitizing local wildlife remains inadequately characterized. This study aimed to (1) investigate tick infestation patterns in wild animals across this ecologically important region, and (2) elucidate the natural enzootic cycles maintaining tick populations in this ecosystem.

## 2 Materials and methods

### 2.1 Sample collection

**Fig. 1:**
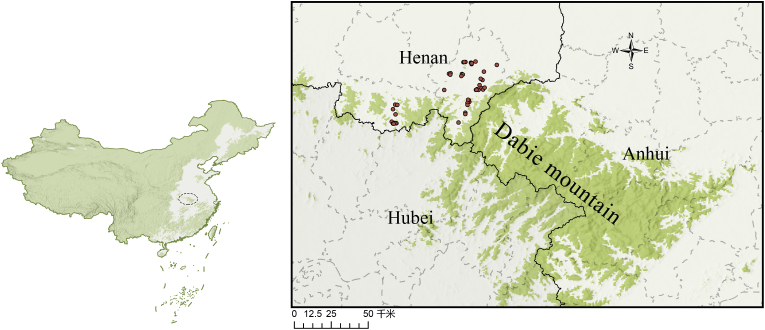
Location of the study area in northern foothills of the Dabie Mountains, China. Sampling sites are marked by orange dots.

**Fig. 2:**
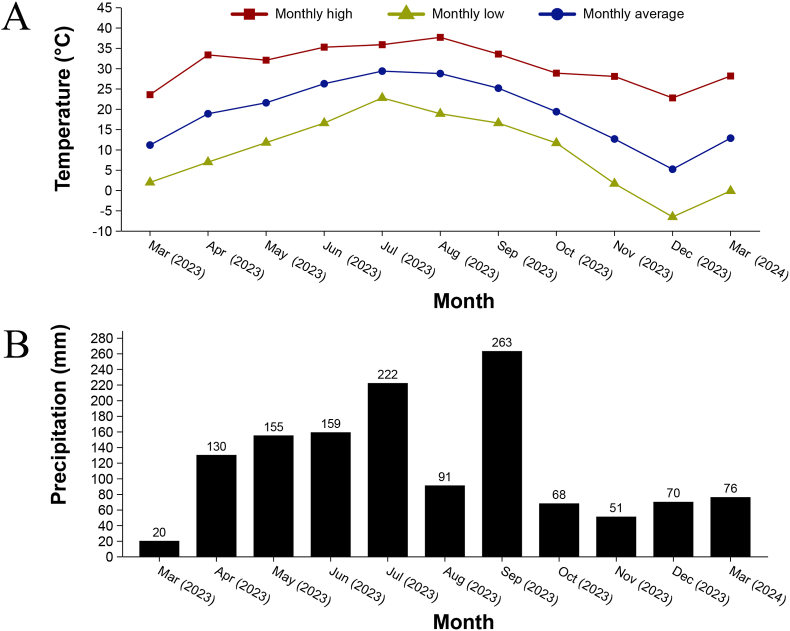
Climate information for the sampling period. A: Monthly temperature; B: Average monthly precipitation. The data are provided by the Chinese Central Meteorological Observatory (https://www.nmc.cn/).

During a comprehensive one-year survey period (March 2023–March 2024), we collected 298 wild mammals across three taxonomic groups in the northern foothills of the Dabie Mountains (31.42°-31.90°N, 114.74°-115.44°E), southeastern Henan Province. The hog badgers were incidentally captured by local residents. For hedgehogs and rodents, wire cage traps baited with peanut butter and dried fruits were deployed in shrubland areas, with ten traps set per site. These traps were checked every morning, and the trapping sessions lasted for 6–7 days. Captured animals were placed in sealed containers and anesthetized using cotton soaked with isoflurane. A full-body examination was then conducted, and ticks were collected using superfine forceps. Additionally, rodents were euthanized and subsequently combed against the fur direction with fine-toothed combs or brushes to gather any dislodged ticks (Machtinger and Williams, 2020). After tick collection, hedgehogs and hog badgers were released back into the wild. The sample comprised 267 rodents (Rodentia), 27 Amur hedgehogs (*Erinaceus amurensis*), and 4 northern hog badgers (*Arctonyx albogularis*), representing key components of the local mammalian fauna. Specimens were collected using standardized trapping protocols across representative habitats within the study region. (Fig. 1). The study region exhibits four distinct meteorological seasons: spring (March–May), summer (June–August), autumn (September–November), and winter (December–February). Our sampling period captured the complete annual climatic cycle, with ambient temperature and precipitation levels showing characteristic seasonal fluctuations (Fig. 2). Specifically, these parameters began increasing during spring sampling, peaked in summer months, and subsequently declined progressively through autumn to winter.

### 2.2 Species identification

Following euthanasia, all specimens underwent systematic ectoparasite examination. Ticks were methodically collected from: (1) sparsely haired anatomical regions including auricles, periorbital areas, labial margins, buccal regions, caudal surfaces, and pedal extremities; and (2) any specimens dislodged from densely haired regions during handling. This comprehensive approach ensured complete sampling of both attached and detached ectoparasites. For hedgehogs and hog badgers, ticks were collected through thorough examination before releasing back to the animals' natural habitat. Initial taxonomic identification of both host animals and ticks was conducted by trained biologists using standard morphological characteristics. To clarify taxonomic ambiguities, we employed molecular confirmation through: (1) partial cytochrome *b* (cytb) gene sequencing for rodent hosts, and (2) small subunit ribosomal RNA (SSU rRNA) gene analysis for tick specimens (Black and Piesman, 1994; Robins et al., 2007). Immature tick stages (larvae and nymphs) were primarily identified through SSU rRNA gene sequencing due to their limited diagnostic morphological features. Genomic DNA extraction was performed using the Universal Genomic DNA Kit (CWBIO, China) according to the manufacturer's protocol. PCR products displaying the expected amplicon size were subjected to bidirectional Sanger sequencing by SinoGenoMax (Beijing, China) to ensure sequence accuracy. All obtained sequences were aligned against reference sequences retrieved from GenBank using ClustalX 2.1 (http://www.clustal.org/), with manual verification of alignment quality.

### 2.3 Statistical analysis

The abundance of each tick species attached to small wild mammals were determined. The number of different tick species on different host animal was compared using the Kruskal–Wallis test by SPSS (v26), and p < 0.05 was considered statistically significant (Umlauft et al., 2017). We examined differences in tick infection rates among hosts using Fisher's exact test (implemented with the fisher.test function in R), followed by post-hoc tests for pairwise comparisons. Differences in tick abundance were assessed by fitting generalized linear models (GLM) with a Negative Binomial distribution, using the MASS package in R. For tick community composition, we employed PERMANOVA (via the vegan package) on community matrices, and visualized patterns with Non-metric Multidimensional Scaling (NMDS) plots generated in ggplot2, based on Bray–Curtis dissimilarities.

## 3 Results

### 3.1 Identification of animal and tick species

A total of 1894 ticks were collected from 267 wild rodents, 27 hedgehogs, and 4 hog badgers (Table s1). Morphological and molecular identification confirmed seven wild rodent species as hosts: *Niviventer lotipes* (n = 117), *Apodemus agrarius* (n = 98), *Rattus nitidus* (n = 29), *Apodemus draco* (n = 18), *Rattus flavipectus* (n = 3), *Microtus mandarinus* (n = 1), and *Arvicola terrestris* (n = 1). Among the 267 captured wild rodents, 31.8 % (85/267) were infested with at least one tick, including 44 *Niviventer lotipes*, 23 *Apodemus agrarius*, 14 *Rattus nitidus*, 2 *Apodemus draco*, and 2 *Rattus flavipectus*. From these infested rodents, we collected 563 ticks total, with the following life stage composition: 50.6 % larvae (285/563), 40.5 % nymphs (228/563), and 8.9 % adults (50/563). Morphological identification revealed two tick species: *H. hystricis* (n = 470) and *I. granulatus* (n = 107). The life stage composition of infesting ticks varied between species. For *H. hystricis*, larvae (n = 282) and nymphs (n = 188) predominated, while *I. granulatus* populations consisted primarily of nymphs (n = 40), followed by adults (n = 50), with only three larvae observed (Table 1).

**Table 1:** Tick species and numbers in small wild mammals.

Species		*N. lotipes*	*A. agrarius*	*R. nitidus*	*A. draco*	*R. flavipectus*	*E. amurensis*	*A.collaris*
*H. hystricis*	Larva	193	3	84	0	2	0	0
	Nymphs	110	7	11	3	57	8	2
	Adults	0	0	0	0	0	8	62
	Subtotal	303	10	95	3	59	16	64
*I. granulatus*	Larva	0	0	3	0	0	0	0
	Nymphs	3	10	27	0	0	0	0
	Adults	21	28	0	1	0	0	0
	Subtotal	24	38	30	1	0	0	0
*H. flava*	Larva	0	0	0	0	0	8	0
	Nymphs	0	0	0	0	0	154	0
	Adults	0	0	0	0	0	967	3
	Subtotal	0	0	0	0	0	1129	3
*H. longicornis*	Larva	0	0	0	0	0	0	0
	Nymphs	0	0	0	0	0	0	1
	Adults	0	0	0	0	0	77	2
	Subtotal	0	0	0	0	0	77	3
*A. testudinarium*	Larva	0	0	0	0	0	0	0
	Nymphs	0	0	0	0	0	39	0
	Adults	0	0	0	0	0	0	0
	Subtotal	0	0	0	0	0	39	0
	Total	327	48	125	4	59	1261	70

**Fig. 3:**
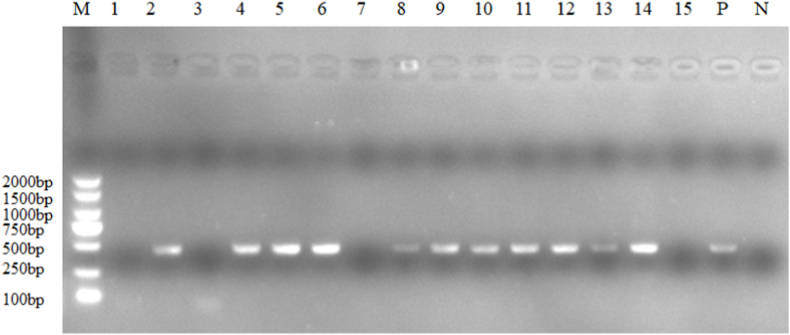
The partial PCR amplification results of ticks. The PCR primers target the mitochondrial 16S rDNA sequence of hard ticks (Ixodidae), with an expected amplicon length of 436 bp.

**Fig. 4:**
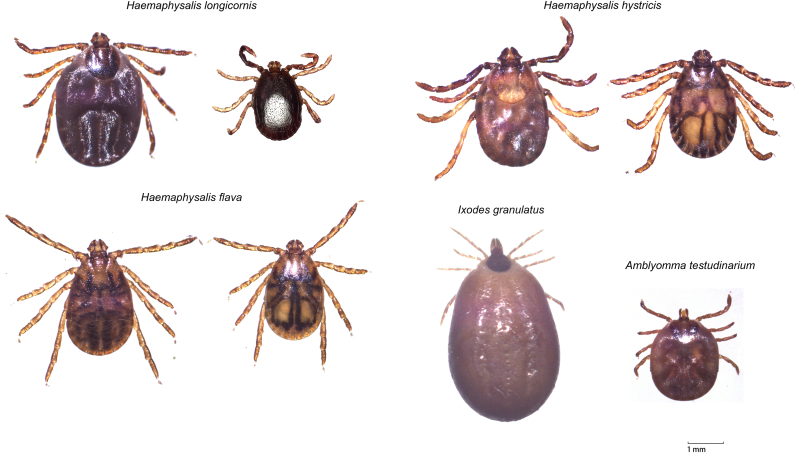
The species and morphology of ticks discovered in this study.

Tick samples were also collected from 27 hedgehogs (*Erinaceus amurensis*), yielding 1261 ticks compromising 2 genera and 4 species: *H. hystricis* (n = 16), *H. flava* (n = 1129), *H. longicornis* (n = 77), and *A. testudinarium* (n = 39). All *H. longicornis* specimens from *E. amurensis* were adults, while *A. testudinarium* occurred exclusively as nymphs. *H. flava* dominated the hedgehog tick community, with adults comprising 85.7 % (967/1129) of specimens, followed by nymphs (13.6 %, 154/1129) and larvae (0.7 %, 8/1129). We also obtained 64 *H. hystricis* (62 adults, 2 nymphs), 3 adult *H. flava*, and 3 *H. longicornis* (2 adults, 1 nymph) from four hog badgers (*Arctonyx collaris*) provided by local farmers (Table 1). Representative tick morphological features and PCR amplification results are presented in Fig. 3, Fig. 4, respectively.

### 3.2 Seasonal variation of ticks in wild small mammals

**Fig. 5:**
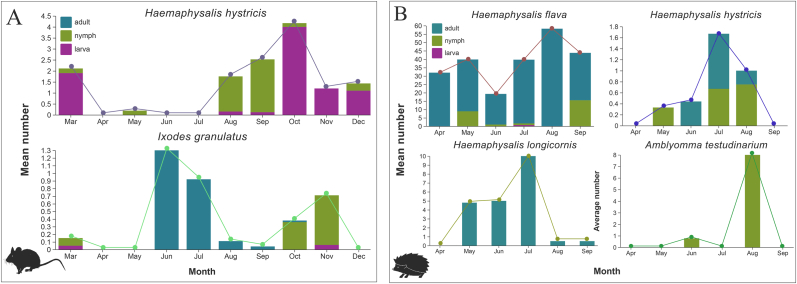
Seasonality of the prevalence of each tick species and their developmental stages parasitizing rodent (A) and hedgehog (B). The line graphs indicate the changes in total number, and the stacked histograms show the composition of different developmental stages.

The infestation patterns of tick species showed distinct seasonal dynamics. For *H. hystricis*, larva and nymph first appeared in March, however, the larva disappears from April to July, and reappears from August to November, which peaking in October. Nymphs were primarily active from May to September, with their peak occurring in July. Adults were mainly active from June to August, also peaking in July (Fig. 5A and B).

*Haemaphysalis flava* was the predominant blood-feeding tick species infesting hedgehogs. The larvae were detected exclusively in July, nymphs demonstrated peak activity during May and September, while adults maintained consistently high infestation levels from March through September, reaching maximal abundance in August. The *H. longicornis* were primarily prevalent during summer, with their initial appearance witnessed in May, reaching the peak abundance in July, and then gradually decrease still September (Fig. 5B).

*Ixodes granulatus* exhibited exclusive parasitism of wild rodents. The larval only found in March and November, and nymph appeared in March, October and November reached, which peaked prevalence in November. Adult ticks demonstrated distinct temporal dynamics, being discovered and achieving maximum infestation rates in June before gradually declining until their complete disappearance by September (Fig. 5A). For *A. testudinarium*, nymphs were detected in summer, first appearing in June and reaching peak infestation levels on hedgehogs in August (Fig. 5B).

### 3.3 Differences in tick infection rates and abundance among hosts

Fisher's exact test with Bonferroni-corrected p-values revealed highly significant differences in infection rates among host genera (P < 0.001), indicating a substantial impact of host genus on infection status. Post-hoc pairwise comparisons (Bonferroni-adjusted) further showed that the infection rate in *Erinaceus* was significantly higher than in *Apodemus*, *Rattus*, and *Niviventer* (P < 0.001) (Table 2).

**Table 2:** Differences in infection rates among different host genera.

Genus	Number infected	Number uninfected	Infection rate
*Apodemus*^a^	20	65	23.53 %
*Rattus*^a^	15	17	46.88 %
*Niviventer*^a^	40	65	38.10 %
*Microtus*^ab^	0	1	0 %
*Arvicola*^ab^	0	1	0 %
*Erinaceus*^b^	27	0	100 %
*Arctonyx*^b^	4	0	100 %

Groups sharing the same letter are not significantly different (*P* > 0.05), whereas groups with different letters differ significantly (*P* < 0.05).

Results from the generalized linear model (GLM) analysis indicated a highly significant overall effect of host genus on tick abundance (χ^2^ = 117.18, P < 0.001). In post-hoc pairwise comparisons conducted with Tukey's method after negative binomial regression, *Apodemus* exhibited significantly lower tick abundance relative to *Erinaceus* (P < 0.001), *Niviventer* (P < 0.001), *Rattus* (P < 0.001), and *Arctonyx* (P < 0.005). Additionally, *Erinaceus* had significantly higher abundance than *Niviventer* (P < 0.001) and *Rattus* (P < 0.001) (Table 3).

**Table 3:** The generalize linear model analyses results.

Contrast	Estimate	Standard error	Z-score	P-value
*Apodemus* vs *Arctonyx*	−3.67	1.00	−3.68	0.0044
*Apodemus* vs *Arvicola*	16.50	3470.00	0.01	1
*Apodemus* vs *Erinaceus*	−4.65	0.44	−10.68	<0.0001
*Apodemus* vs *Microtus*	16.50	3470.00	0.01	1
*Apodemus* vs *Niviventer*	−1.83	0.29	−6.24	<0.0001
*Apodemus* vs *Rattus*	−2.55	0.42	−6.14	<0.0001
*Arctonyx* vs *Arvicola*	20.17	3470.00	0.01	1
*Arctonyx* vs *Erinaceus*	−0.98	1.04	−0.94	0.9653
*Arctonyx* vs *Microtus*	20.17	3470.00	0.01	1
*Arctonyx* vs *Niviventer*	1.83	0.99	1.86	0.5098
*Arctonyx* vs *Rattus*	1.11	1.03	1.08	0.9343
*Arvicola* vs *Erinaceus*	−21.15	3470.00	−0.01	1
*Arvicola* vs *Microtus*	0.00	4900.00	0.00	1
*Arvicola* vs *Niviventer*	−18.33	3470.00	−0.01	1
*Arvicola* vs *Rattus*	−19.05	3470.00	−0.01	1
*Erinaceus* vs *Microtus*	21.15	3470.00	0.01	1
*Erinaceus* vs *Niviventer*	2.82	0.42	6.77	<0.0001
*Erinaceus* vs *Rattus*	2.10	0.51	4.11	0.0008
*Microtus* vs *Niviventer*	−18.33	3470.00	−0.01	1
*Microtus* vs *Rattus*	−19.05	3470.00	−0.01	1
*Niviventer* vs *Rattus*	−0.72	0.40	−1.83	0.5306

### 3.4 Host preference of ticks in wild small mammals

**Fig. 6:**
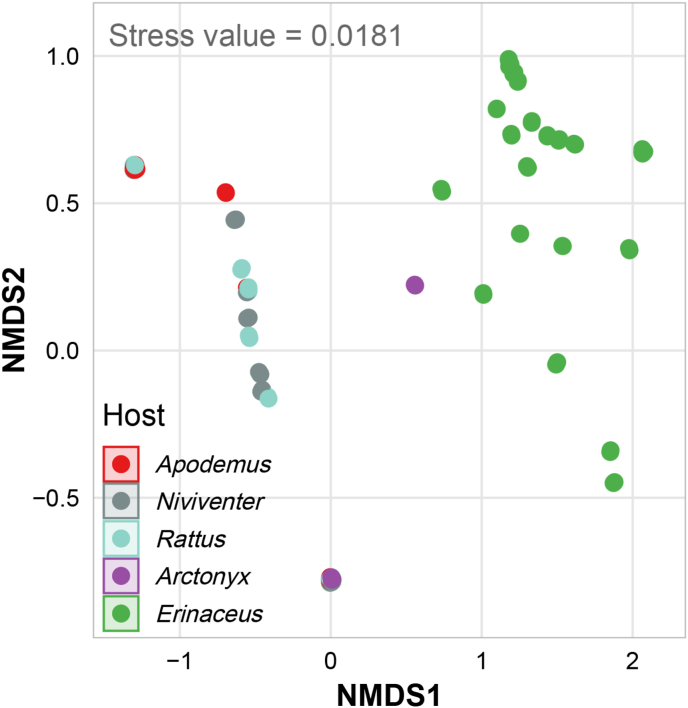
Non-metric multidimensional scaling (NMDS) plot of tick community composition based on Bray–Curtis distances.

PERMANOVA revealed significant differences in tick community composition among host species (F = 20.787, R^2^ = 0.430, *P* = 0.001). The NMDS plot further illustrated distinct clustering patterns: *Erinaceus* harbored a unique tick assemblage, while the rodent hosts (*Apodemus*, *Niviventer*, and *Rattus*) shared similar community compositions. Some individual rodents and hog badger also showed overlapping tick communities, whereas hedgehog clearly separated from all other host groups (Fig. 6).

**Fig. 7:**
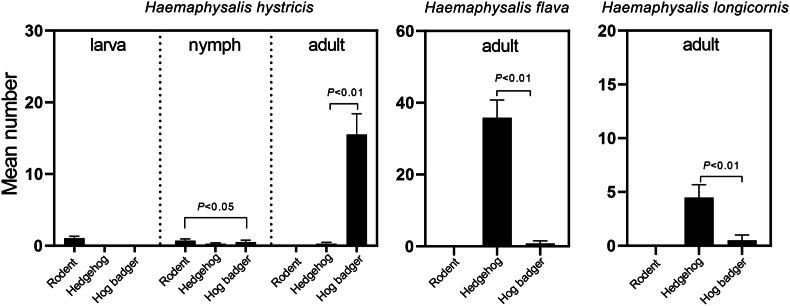
Host selectivity of ticks at different developmental stages in small wild mammals. Error bars represent standard errors of the mean number of ticks attached to each animal.

*Haemaphysalis hystricis* was found in all three host species; its larvae exclusively parasitized rodents, nymphs being detected in all three host animals but with significantly higher number on rodents compared to hog badgers (*P* < 0.05), while adults primarily infested hedgehogs and hog badgers, with a notable abundance on hog badgers (*P* < 0.01). With regard to *H. flava*, larvae and nymphs were found only on hedgehogs, while adults parasitized both hedgehogs and hog badgers but with significantly higher burden on hedgehogs. Similarly, *H. longicornis* nymphs were exclusively detected on hog badgers, and adults on hedgehogs and hog badgers, with significantly higher number on hedgehogs (Fig. 7).

**Fig. 8:**
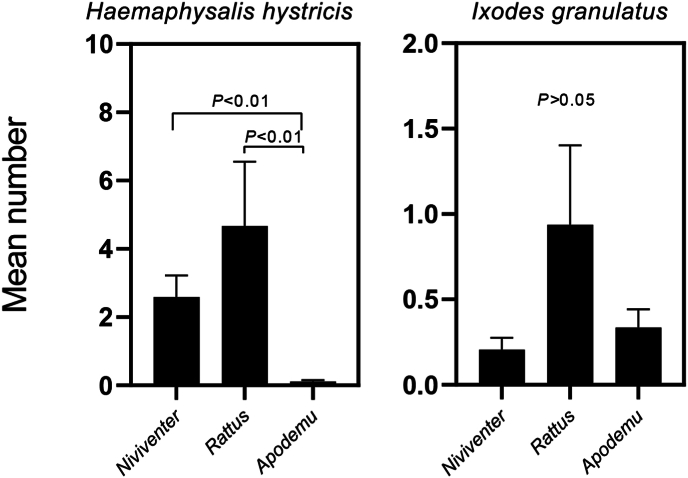
Host selectivity of ticks in rodents. Error bars represent standard errors of the mean number of ticks attached to each rodent.

Ticks also show different host preferences among rodents. The *H. hystricis* were found on three genera of rodents (*Niviventer*, *Rattus* and *Apodemus*), which had a significantly higher number on *Niviventer* and *Rattus* compared to *Apodemus* (*P* < 0.01). However, *I. granulatus* was found to be demonstrating strong host specificity owing to the presence of its all life stages on the surface of rodents, which were not observed to differ significantly among the three genera (*P* > 0.05) (Fig. 8). The other species, nymphs of *A. testudinarium*, were only observed on hedgehogs, with no other life stages collected.

## 4 Discussion

Small wild mammals play significant roles in maintaining tick life cycles and facilitating the dispersal of both ticks and their associated pathogens. Our investigation focused on tick infestations across three prevalent small mammal species inhabiting the northern foothills of China's Dabie Mountains. A total of five species of ticks were identified - *H. hystricis*, *H. flava*, *H longicornis.*, *I. granulatus*, and *A. testudinarium*. Among them, *H. longicornis* and *H. flava* are common tick species in this region and have already been documented several times (Zhao et al., 2022; Zhuang et al., 2018). *Haemaphysalis longicornis* is the primary vector for SFTSV and has been responsible for outbreaks of this zoonotic tick-borne disease in local communities (Yu et al., 2011a), Additionally, pathogens such as *Anaplasma* spp., *Rickettsia* spp. and *Babesia* sp. have been detected in local *H. longicornis* populations (Zhuang et al., 2018). In China, *H. flava* has been documented to carry multiple pathogens, including *Anaplasma* spp., *Rickettsia* spp., *Coxiella burnetii*, Jingmen tick virus and SFTSV (Fang et al., 2023; Zhao et al., 2021). However, comprehensive studies on the full spectrum of pathogens carried by *H. flava* in local settings remain limited. *Haemaphysalis hystricis*, *I. granulatus*, and *A. testudinarium* have been reported in other regions of China, our study represents their first recorded occurrence in this specific geographic area. *Haemaphysalis hystricis* is widely distributed across central and southwestern China and has been found to harbor multiple pathogens, including and *Rickettsia* spp., Tacheng tick virus, Wenzhou tick virus, Yongjia tick virus and Jingmen tick virus (Lu et al., 2017; Shu et al., 2024; Zhao et al., 2021; Zheng et al., 2019). *Ixodes granulatus* is primarily documented in southern China, while the *A. testudinarium* is frequently reported in southwestern and southeastern regions. Both species have been found to carry *Anaplasma* spp., *Borrelia* spp. and Jingmen tick virus (Yang et al., 2021; Zhang et al., 2022c). The five tick species identified in this study are all capable of carrying multiple pathogens, underscoring the need for further research into their potential impact on local public health safety.

Tick infestation abundance on small wild mammals exhibits distinct seasonal patterns. Based on the previous field observations, ticks detected on small wild mammals were found to make their first appearance in March, with abundance peaking in summer and autumn before declining sharply in winter, which is a common phenomenon (Meng et al., 2016; Yu et al., 2011b; Zheng et al., 2012). Ectoparasite loads exhibited marked seasonal fluctuations, with temperature and humidity emerging as critical abiotic drivers governing tick survival and activity patterns (Hauck et al., 2020; Probst et al., 2023; Sands et al., 2021). In the present study region, spring and autumn average temperature consistently exceed 10 °C, and the average temperature remains between 20 °C and 25 °C in summer, with abundant precipitation. In contrast, winter temperatures frequently drop below 10 °C, often reaching subzero levels (Bansal et al., 2021; Gethmann et al., 2020). Moreover, under elevated temperatures, relative humidity emerges as an increasingly critical determinant of tick viability, demonstrating a positive association with survival probability (Sands et al., 2021). Within lower thermal ranges (4–8 °C), certain ixodid species maintain developmental capability, albeit with significantly reduced growth rates (p < 0.05) and elevated mortality indices (Bansal et al., 2021).

Ticks demonstrate species- and stage-specific host preferences. Our study revealed that *I. granulatus* primarily infests rodent hosts, completing its entire life cycle on small wild mammals (Kwak et al., 2023; Shih et al., 2021; Zheng et al., 2019), while demonstrating occasional opportunistic feeding on livestock and humans (Paperna, 2006). Furthermore, our study identified nymphs of *A*. *testudinarium* exclusively on hedgehogs, while adults predominantly parasitized medium-to-large mammals and frequently engaged in human biting behavior (Islam et al., 2006; Nakamura-Uchiyama et al., 2000; Yamauchi et al., 2013). Three *Haemaphysalis* species (*H. hystricis*, *H. flava*, and *H. longicornis*) also exhibited distinct primary host preferences. Consistent with earlier reports, *H. hystricis* was observed to complete its life cycle on small wild animals, with frequent infestations of wild boar and livestock, and occasional human bites (Chiang et al., 2024; Lu et al., 2017). *Haemaphysalis flava* was primarily found on hedgehogs and hog badgers in this study. Although it parasitizes wild animals broadly, hedgehogs are its most common host in China (Qi et al., 2022; Wang et al., 2024). *Haemaphysalis longicornis* emerged as the dominant tick species in the study area. Previous research noted that all its developmental stages more frequently infest livestock (Zheng et al., 2012). While larvae and nymphs of multi-host hard ticks generally prefer small mammals (McCoy et al., 2013), and *H. longicornis* was not the dominant species among local wild small mammals—a pattern also reported in other studies of small wild mammals in China (Fang et al., 2021; Qi et al., 2022; Zheng et al., 2012).

## 5 Conclusion

This study documents three genera (*Haemaphysalis*, *Ixodes*, *Amblyomma*) comprising five tick species parasitizing small wild mammals in the northern foothills of the Dabie Mountains, revealing critical behavior patterns regarding their seasonality and host preferences. Notably, we report the first recorded presence of *H. hystricis*, *I. granulatus*, and *A. testudinarium* in this region, represents a major expansion of their known distribution ranges. Our findings demonstrate that tick abundance follows distinct seasonal dynamics, strongly correlated with both species and developmental stage. We further reveal that certain tick species exhibit pronounced wildlife host associations, suggesting potential hotspots for zoonotic disease transmission. By characterizing the seasonal diversity and stage-specific host selection behaviors of these five tick species across multiple small mammal hosts, our work provides a foundation for future investigations of tick ecology in changing environments. The five tick species identified in this study are all capable of carrying multiple pathogens, therefore, systematic monitoring of their diversity, host specificity, and distribution patterns—particularly in response to climate change and land-use modifications—is essential for predicting and mitigating public health risks.

## CRediT authorship contribution statement

**Yin Fu:** Writing – review & editing, Writing – original draft, Visualization, Validation. **Pitambar Dhakal:** Writing – review & editing, Investigation. **Zi Yan:** Writing – review & editing, Investigation. **Mengyao Yang:** Writing – review & editing, Investigation. **Chaofeng Ma:** Writing – review & editing, Investigation. **Yayun Wu:** Writing – review & editing, Investigation. **Jiahui Wang:** Writing – review & editing, Investigation. **Qinglin Wang:** Writing – review & editing, Investigation. **Longxian Zhang:** Writing – review & editing, Supervision, Methodology, Funding acquisition, Conceptualization.

## Funding

This work was supported in part by the Henan Province Key Research and Development Program (231111111500 to LZ), the 10.13039/501100012166National Key Research and Development Program of China (2022YFD1800200 to LZ), and the 10.13039/501100001809National Natural Science Foundation of China (U1904203 to LZ).

## Conflict of interest

The authors declared that the research was conducted in the absence of any commercial or financial relationships that could be construed as a potential conflict of interest. Also, we have no personal relationships conflict of interest in this work.

## Data Availability

Data will be made available on request.
